# Characterization of Transparent Fluorapatite Ceramics Fabricated by Spark Plasma Sintering

**DOI:** 10.3390/ma15228157

**Published:** 2022-11-17

**Authors:** Hiroaki Furuse, Daichi Kato, Koji Morita, Tohru S. Suzuki, Byung-Nam Kim

**Affiliations:** 1 Kitami Institute of Technology, 165 Koen-cho, Kitami 090-8507, Hokkaido, Japan; 2 National Institute for Materials Science, 1-2-1, Sengen, Tsukuba 305-0047, Ibaraki, Japan

**Keywords:** fluorapatite, transparent ceramics, spark plasma sintering, fine microstructure

## Abstract

Highly optically transparent polycrystalline fluorapatite ceramics with hexagonal crystal structures were fabricated via a liquid-phase synthesis of fluorapatite powder, followed by spark plasma sintering (SPS). The effect of sintering temperature, as observed using a thermopile, on the optical transmittance and microstructure of the ceramics was investigated in order to determine suitable sintering conditions. As a result, high optical transmittance was obtained in the SPS temperature range of 950–1100 °C. The highest optical transmittance was obtained for the ceramic sample sintered at 1000 °C, and its average grain size was evaluated at only 134 nm. The grain size dramatically increased with temperature, and the ceramics became translucent at SPS temperatures above 1200 °C. The mechanical and thermal properties of the ceramics were measured to evaluate the thermal shock parameter, which was found to be comparable to or slightly smaller than that of single-crystal fluorapatite. This transparent polycrystalline fluorapatite ceramic material should prove useful in a wide range of applications, for example as a biomaterial or optical/laser material, in the future. Furthermore, the knowledge obtained in this study should help to promote the application of this ceramic material.

## 1. Introduction

The apatite group of minerals has the stoichiometry A_10_(XO_4_)_6_Z_2_ and a hexagonal crystal structure, and this group spans a wide range of compositions with A = Ca, Sr, Ba, etc., X = P, V, etc., Z = OH, F, Cl, etc. Hydroxyapatite (Ca_10_(PO_4_)_6_(OH)_2_: HAP) in particular has been widely studied as a representative biomaterial such as artificial bone or teeth because of its high biocompatibility [1,2]. Furthermore, translucent HAP ceramics have been realized using various fabrication processes; for example, research on cell culture scaffolds made from this material has been reported [3,4,5]. In recent years, investigations into the biocompatibility of fluorapatite (Ca_10_(PO_4_)_6_F_2_, FAP), in which OH ions in the HAP structure are replaced by F ions, has advanced [6,7,8].

As well as biomaterials applications, fluorapatite is considered an important laser material, despite the fact that HAP is not a phosphor, because of the strong quenching due to energy transfer between the rare earth (RE) and OH ions [9]. In particular, Yb-doped Sr_10_(PO_4_)_6_F_2_ (Yb:S-FAP) has a higher stimulated emission cross section than yttrium aluminum garnet (YAG), and hence it is used as a high-power laser material [10,11,12]. In fact, single-crystal Yb:S-FAP produced a 60-J, 10-Hz high-energy laser system in 2003 [13]. In recent years, polycrystalline cubic Yb:YAG ceramics have come to be considered representative high-energy laser materials, and output energies of >100 J with a 10-Hz repetition rate have been reported [14,15]. Such achievements have occurred owing to the establishment of manufacturing technology for high-quality large-area YAG ceramics. For the development of high-energy laser technology, the preparation of laser materials with large cross-sectional areas is necessary to prevent surface damage to the crystal by allowing increased laser beam cross-sectional areas and reduced laser fluence. Furthermore, in order to suppress the parasitic lasing (PL) that typically occurs in the radial direction, which is noticeable for large-diameter crystals, bonding an absorber to the laser material frame is indispensable. In these respects, ceramic manufacturing technology has great advantages over single-crystal manufacturing technology. Therefore, it is expected that even higher laser outputs will be achieved if transparent FAP or S-FAP ceramics are realized.

Since apatite is a uniaxial crystal, the in-line transmittance of the bulk polycrystalline ceramic material is generally low because of grain boundary scattering, even when as many as possible of the residual pores, which are the main scattering source for ceramics, are eliminated. The amount of grain boundary scattering *γ* is depends on the average grain size *d*, average refractive index difference at the grain boundary Δ*n*, and the wavelength of light *λ* as *γ* = 3π^2^*d*Δ*n*^2^/2*λ*^2^, if the grain size is less than a micrometer [16]. Thus, the preparation of nano-grained and fully dense hydroxyapatite (HAP) ceramics has been reported via ultra-high-pressure sintering [17], hot-isostatic pressing (HIP) [18,19], and spark plasma sintering (SPS) [20,21,22,23,24,25,26,27]. However, there are few studies on transparent FAP ceramics, despite the fact that these are important because of their potential to be applied as both laser materials and biomaterials.

By using SPS to control the crystal grain size to less than the wavelength of light and reduce grain boundary scattering, we have been able to develop transparent Nd- and Yb-doped FAP ceramics with laser-grade optical quality. In addition, we succeeded in demonstrating their laser oscillation, even though they were composed of randomly oriented crystal grains [28,29]. However, slight amounts of scattering sources remained in the material, and the laser’s efficiency was low. In order to use apatite ceramics with a fine microstructure as an effective laser material, it is necessary to achieve an optical quality equivalent to that of single crystal laser materials and to realize higher laser output power. In addition, the fundamental physical properties, such as the thermal conductivity, thermal expansion coefficient, and mechanical strength of the ceramics, are important for thermal analyses and for the evaluation of the thermal shock parameter, which allows an analysis of the performance of this material compared to single crystal.

In this study, we fabricated transparent FAP ceramics using various SPS temperatures to investigate their sintering behavior. The optical transmittance and microstructures were characterized for each SPS temperature. In addition, the infrared absorption of the material was measured in order to determine the degree of fluorine substitution. Furthermore, the thermal conductivity, thermal expansion, and mechanical strength of the ceramic sample with the highest transmittance were characterized.

## 2. Materials and Methods

### 2.1. Sample Preparation

**Figure 1:**
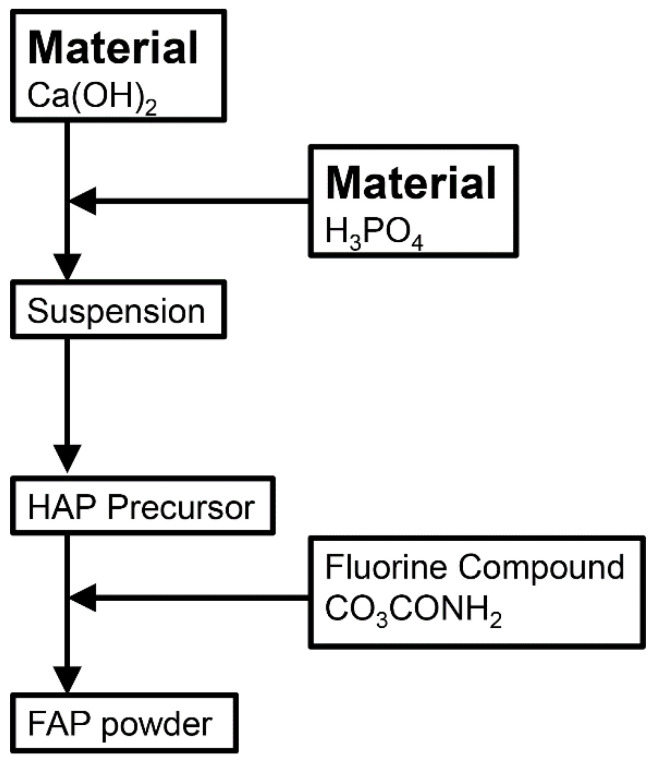
Schematic diagram of the synthesis of the FAP powder.

Figure 1 shows the outline of the fabrication process for the FAP powder, which was used to prepare the final FAP ceramic sample. Calcium hydroxide and phosphoric acid were used as the starting materials; these were mixed to synthesize a HAP precursor. An aqueous solution of trifluoroacetamide (CF_3_CONH_2_) was mixed with the HAP precursor and then heated at 600 °C for 2 h to replace the hydroxide ions in the HAP with fluorine ions to obtain a FAP powder.

The sieved powder was sintered using an SPS machine (LABOX-315, Sinter Land, Niigata, Japan) to obtain dense ceramics. Specifically, the powder was poured into a graphite mold with a 10-mm inner diameter and uniaxially pressed using a graphite punch. A carbon sheet was placed between the powder and the sintering mold and punches. The temperature was measured on the surface of the graphite mold using an optical pyrometer. The SPS temperature was varied between 900 and 1200 °C in intervals of approximately 50 °C to investigate its effect on the optical properties and grain growth of the ceramics. In each case, the heating rate was set to 5 °C/min, the uniaxial applied pressure was approximately 80 MPa, and the holding time was 20 min. Furthermore, the temperature was lowered at a rate of 10 °C/min to prevent sample cracking. After sintering, both surfaces of each sample were mirror-polished using a 1 μm diamond slurry to a thickness of ~1.3 mm in preparation for the optical transmittance measurement and microstructure observation. For the thermal conductivity and mechanical strength measurements, samples of the FAP ceramics with 15-mm diameters were prepared using the same fabrication process before being cut to appropriate sizes (see Section 2.3).

### 2.2. Optical, Chemical Content, and Microstructure Characterization

The optical in-line transmittance spectra of the specimens were measured using an UV/Vis/NIR spectrometer (SolidSpec-3700i DUV, Shimadzu, Kyoto, Japan). To compare the optical transmittance of specimens of different thicknesses, we evaluated the total loss coefficient *δ*, including both the absorption and scattering losses, from the optical transmittance spectrum using:

(1) $$T={\left(1-R\right)}^{2}\exp\left(-\delta L\right),$$

where *R* is the theoretical reflectance obtained from the refractive index dispersion *n* of the undoped FAP single crystal [30] as *R* = (1 − *n*)^2^/(1 + *n*)^2^ and *L* is the sample thickness, which was measured using a micrometer screw gauge. From the loss coefficient for each specimen, we estimated the transmission spectrum for a thickness of 1.0 mm, *T*.

The crystal structures of FAP powders and transparent ceramics were characterized using X-ray diffraction (XRD; Ultima IV, Rigaku, Tokyo, Japan). The microstructures of FAP powders and all the ceramics were observed using field-emission scanning electron microscopy (FE-SEM; JSM-6701F, JEOL, Tokyo, Japan). Prior to the FE-SEM measurements, the polished surfaces of each sample were thermally etched for 1 h at a temperature 100 °C lower than that used for sintering it. The average grain sizes were determined from the acquired FE-SEM images, using approximately 150 grains for all the specimens.

The infrared transmitted spectra of FAP ceramics were also characterized by another spectrometer (UV-3100PC, Shimadzu, Kyoto, Japan) and a Fourier transform infrared spectrometer (FT-IR; FT/IR-660Plus, JASCO, Tokyo, Japan) to confirm that fluorine substitution had occurred. For comparison, the infrared transmittance spectrum was also measured for the HAP ceramics produced without mixing with the fluorine compound. For the HAP ceramics, the SPS conditions used were the same as those reported in Ref. [29].

### 2.3. Physical Properties

Fundamental physical properties, including thermal conductivity *Κ*, thermal expansion coefficient *α*, and bending strength *σ*, were measured for the ceramic sample sintered at 1000 °C because it was shown to possess the highest in-line transmittance. To evaluate *Κ*, which can be expressed as $K=C_{p}\cdot \rho \cdot \beta$, the specific heat capacity *C_p_*, density *ρ*, and thermal diffusivity *β*, were measured for a FAP ceramics with diameter of 6 mm and thickness of 1 mm. For the *C_p_* and *β* measurements, differential scanning calorimetry (DSC8000, PerkinElmer, Waltham, MA, USA) and laser flash analysis (LFA467, Netzsch, Selb, Germany) were used, respectively. The density of the sample was found to be 3.16 g/cm^3^. For the *α* measurements, thermomechanical analysis (TD5020SE, Netzsch, Germany) was used to determine the thermal expansion rate between 30 and 100 °C of a 10-mm-long sample of the FAP ceramics in air with a heating rate of 5 °C/min. For the measurement of *σ*, a universal testing machine (5582, Instron, USA) was used. The four-point bending test was performed for a FAP ceramic (1.15 mm height × 5.00 mm width × 10 mm length) in air with a crosshead speed of 0.5 mm/min. These experiments were performed by the Japan Fine Ceramics Center (JFCC). From these results, the thermal shock parameter *R_T_* of the ceramic material was evaluated as:

(2) $$R_{T}=\frac{\sigma K(1-\nu )}{\alpha E},$$

where *ν* = 0.4 is Poisson’s ratio and *E* = 109 GPa is Young’s modulus, respectively, for the Yb:S-FAP single crystal [13].

## 3. Results and Discussion

**Figure 2:**
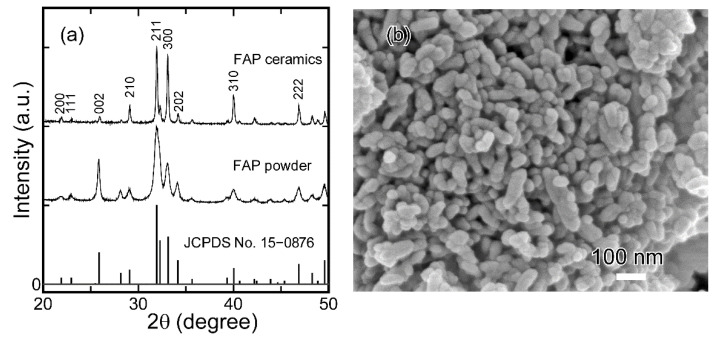
(**a**) XRD pattern of FAP powder and the FAP ceramic material sintered at 1000 °C. (**b**) FE-SEM image of the FAP powder.

Figure 2 shows the normalized XRD pattern and an FE-SEM image of the FAP initial powder. In Figure 2a, the XRD pattern of a FAP ceramic sample sintered at 1000 °C is also shown. The diffraction peaks of the powder are in good agreement with those of the standard diffraction pattern of FAP (JCPDS No. 15-0876), confirming that this sample consists of a single phase. The average crystallite size in the powders was evaluated to be in the range of 15–30 nm. From Figure 2b, it can be seen that the sample apparently consists of fine spherical/elliptical particles, and the particle size appears to be similar to the abovementioned estimated crystallite size.

**Figure 3:**
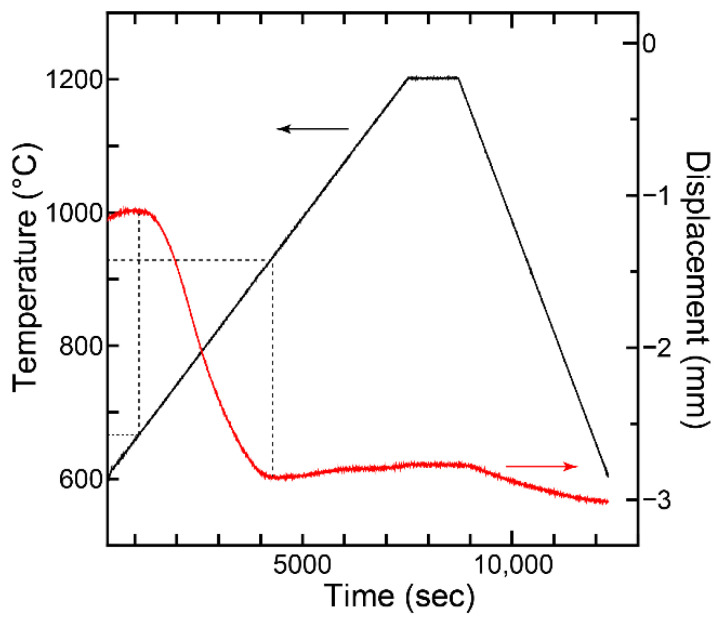
SPS temperature and displacement versus time for FAP ceramics during sintering at 1200 °C.

Figure 3 shows the displacement profile for the FAP ceramic sintering process performed at 1200 °C. It was found that shrinkage started at approximately 680 °C and densification was complete at approximately 920 °C, respectively. A slight thermal expansion was observed subsequent to the densification as the SPS temperature increased. This result suggests that the temperature condition for full densification owing to the removal of pores is in the temperature range exceeding 920 °C.

**Figure 4:**
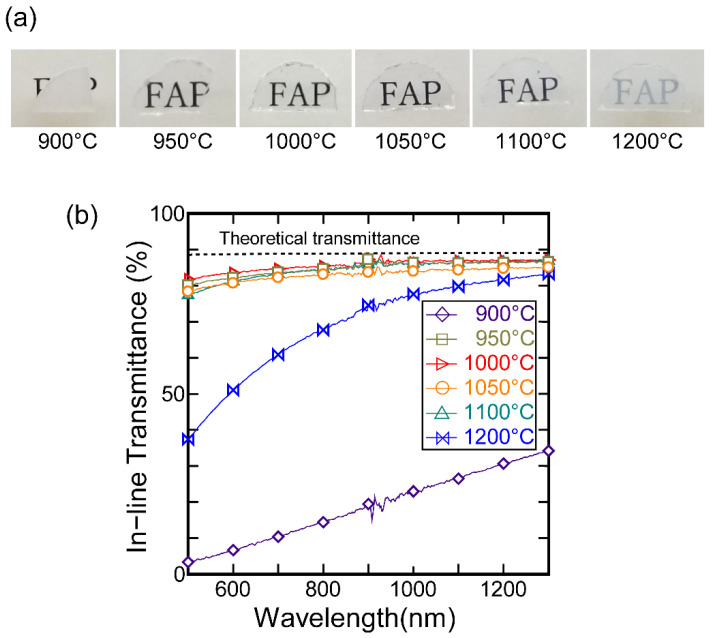
(**a**) Photographs of FAP ceramics sintered at various temperatures. The ceramics were placed 10 mm above the text in each case. (**b**) In-line transmittance spectra of the FAP ceramics.

Figure 4a shows photographs of the FAP specimens sintered at various temperatures, and Figure 4b shows the in-line transmittance spectra in the Vis-NIR region. The photographs in Figure 4a were acquired with the ceramics placed at a position 10 mm above the text. The ceramic samples sintered in the temperature range of 950–1100 °C exhibit high optical transmittance, and the transmittance spectra are very similar, with the exception of that of the sample sintered at 1050 °C. As shown in Table 1 for the in-line transmittance at a wavelength of 1 μm, for three of the samples, >86% optical transmittance was obtained, which is comparable to the Yb:FAP ceramics we have succeeded in lasing in ref. [29]. The highest optical transmittance at *λ* = 1 μm of 86.4%, corresponding to a total loss coefficient of 0.29 cm^−1^, was obtained for the sample prepared using an SPS temperature of 1000 °C. However, the FAP ceramics sintered at 900 and 1200 °C were opaque and translucent, respectively.

**Figure 5:**
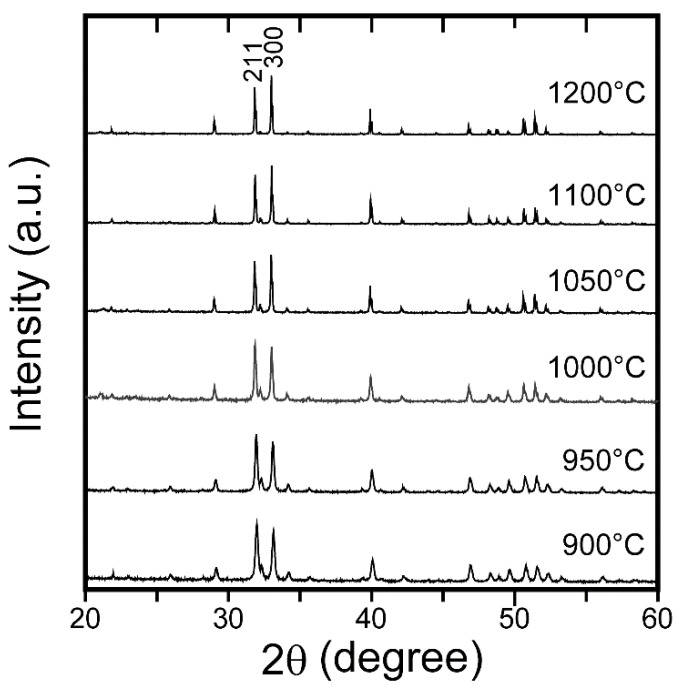
XRD pattern of FAP ceramics sintered at various SPS temperatures.

The XRD diffraction pattern of the FAP ceramics (Figure 2a) consists of peaks with diffraction angles that are in good agreement with those of the standard FAP powder, indicating that the ceramics consist of almost randomly oriented crystal grains. However, the relative peak intensities are slightly different. The intensity of the (002) diffraction peak of the ceramics is lower than that of the powder, whereas the (300) diffraction peak is more intense for the ceramic sample. These differences have been explained by Watanabe et al., who stated that during sintering, the *c*-axis of the crystal grains was aligned perpendicular to the pressure direction [24]. The XRD diffraction patterns of FAP ceramics prepared using different SPS temperatures are shown in Figure 5. The (002) peak intensity decreased as the SPS temperature increased. In addition, by comparing the (211) peak intensities at 31.9° and the (300) peak intensities at 33.1°, it can be observed that the (300) peak intensity increased with SPS temperature. We attribute this to the growth of crystal grains oriented by uniaxial compression. This behavior is in good agreement with that reported by Li et al., in which it is reported that the degree of orientation will be high with the powder having an anisotropic shape [25].

**Figure 6:**
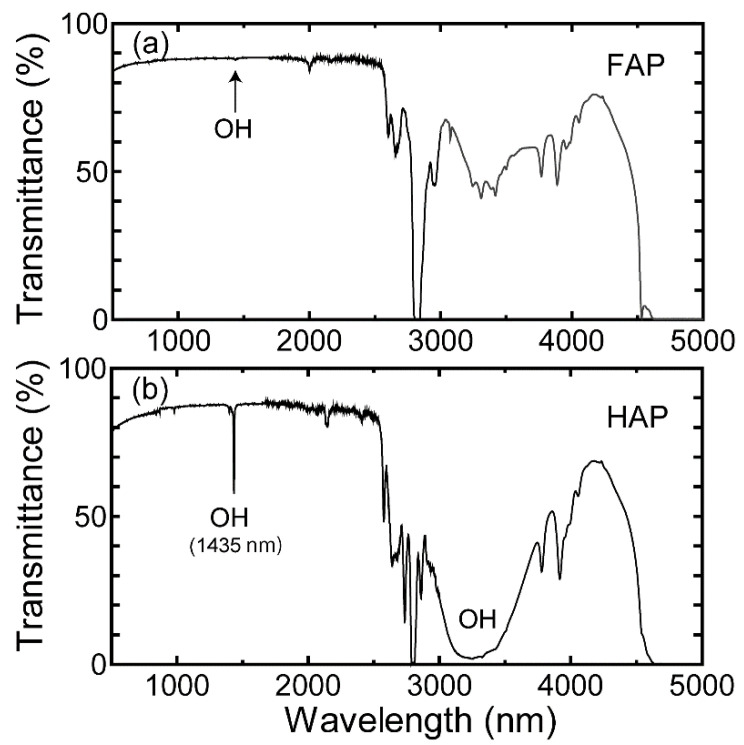
Infrared transmittance spectra of (**a**) FAP and (**b**) HAP ceramics as measured by vis-NIR and FT-IR spectrometry.

Figure 6 shows the infrared transmittance spectra of the FAP and HAP ceramics. The intensity of the absorption band assigned to the first overtone of O–H stretching vibration, at a wavelength of 1435 nm (~7000 cm^−1^), was dramatically reduced for the FAP ceramics. In our previous study of Yb:FAP laser ceramics, an absorption band assigned to residual HAP was observed at 910 nm [29]. Indeed, in Figure 6a, a weak OH absorption band can be seen, as indicated by the arrow. If residual OH remains, it may lead to quenching owing to energy transfer to OH, which may greatly deteriorate the usability of the ceramics as laser materials or phosphors. In order to eliminate this problem, we believe that it is necessary to find an optimize the fluorine replacement method, for example by tuning the amount of fluorine compound used or the heat treatment conditions.

**Figure 7:**
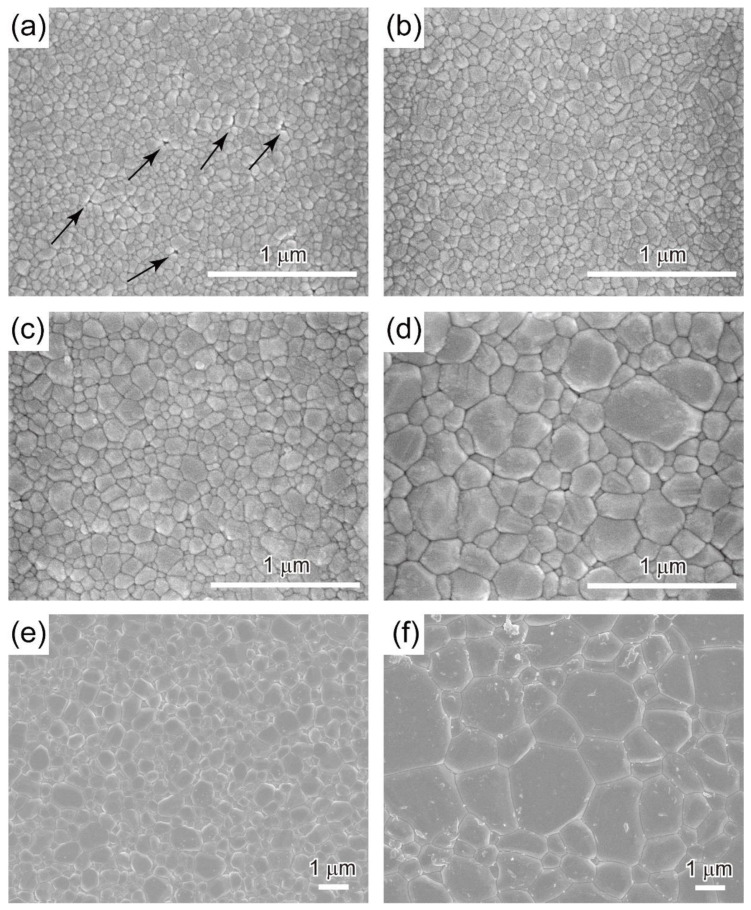
FE-SEM images of the polished surfaces of FAP ceramics sintered at (**a**) 900 °C, (**b**) 950 °C, (**c**) 1000 °C, (**d**) 1050 °C, (**e**) 1100 °C, and (**f**) 1200 °C.

Figure 7 shows the microstructures of the samples sintered at each temperature. The ceramics sintered at 900 °C had the smallest grain size (88 nm); however, some residual pores remained in this sample, as indicated by the overlaid arrows. This is the main reason why the sample was opaque (Figure 4a). At sintering temperatures above 950 °C, no residual pores were observed in the FE-SEM images of the ceramics, which were highly transparent. As shown in Table 1, the average grain size increased dramatically with SPS temperature. In the transmittance spectra shown in Figure 4, no significant difference can be seen between the FAP ceramics sintered at 950 and 1100 °C, even though the crystal grain size differed significantly. For the sintering temperature of 1200 °C, the average grain size in the resultant ceramics was evaluated to be approximately 1.6 μm, and the ceramics appear translucent. The degradation of optical transmittance is under investigation in detail. We attribute it to grain boundary scattering due to birefringence since it is proportional to the average grain size [16] and/or an extremely small number of residual pores that enlarged together with the crystal grains and could not be observed in the FE-SEM image.

Table 2 lists the physical properties of the FAP ceramics sintered at 1000 °C along with the thermal shock parameter [Equation (2)]; values for Poisson’s ration and Young’s modulus of 0.4 and 109 GPa, respectively, were used for the evaluation of *R_T_*. The thermal expansion coefficient (*α*) of the FAP ceramics was 11.5 ppm/K, which is about 15% higher than those of Yb:FAP and S-FAP single crystals [12,31,32]. In cubic laser materials, there is no significant difference in *α* between the polycrystalline ceramics and single-crystal forms [33]. The reason for the discrepancy is under investigation. The thermal conductivity was estimated to be 1.3 W/mK, which is lower than that of single crystals. Since the thermal conductivity is proportional to the mean free path of phonons, the thermal conductivity tends to be low in ceramics with fine microstructures. However, the bending strength was found to be relatively high (161 MPa).

The thermal shock parameter of Yb:S-FAP single crystals was evaluated, assuming an average crack of approximately 25 μm, in Ref. [12] as 125 W/m. The fracture toughness values of the Yb:S-FAP [12] and FAP single crystals [31] are very similar, at 0.51 and 0.48 MPa/m^1/2^, respectively, and we expect that the thermal shock parameter of single-crystal Yb:FAP is comparable to that of S-FAP.

For the FAP ceramics, the thermal shock parameter was evaluated at 100 W/m and there was no significant difference between these parameters for the polycrystalline ceramics and single crystal. Moreover, since mechanical strength tends to be greater for ceramics compared to single crystal because of the abundance of grain boundaries, it is expected that transparent FAP ceramics will be particularly effective as biomaterials, such as artificial bones and tooth materials.

There are some reports on the use of transparent S-FAP ceramics as laser materials [34,35,36,37]; however, laser oscillation has not been reported to the best of our knowledge. This fact suggests that it is more difficult to obtain high-optical-quality transparent S-FAP ceramics capable of laser oscillation. In future studies, we plan to fabricate rare-earth doped S-FAP ceramics using similar synthesis techniques to try to realize lasing and study their basic physical properties with these materials.

## 4. Conclusions

In conclusion, we reported a detailed investigation of the correlation between the SPS temperature and properties—such as optical quality and microstructure—of fluorapatite ceramics, which are expected to be applied as new laser and bio-materials. From the SPS displacement profile, it was apparent that densification was complete at a sintering temperature of 920 °C, as measured using a thermopile. High optical quality transparent ceramics with typical in-line transmittance of >86% were obtained in the SPS temperature range of 950–1100 °C. The highest quality ceramic material was obtained at 1000 °C, and the average grain size of the ceramics was 134 nm. We measured the thermal expansion coefficient, thermal conductivity, and bending strength of this ceramic material and estimated the thermal shock parameter, which was found to be comparable to that of single crystal FAP.

In future studies, we plan to perform similar characterization of rare-earth doped FAP ceramics to determine the optimum sintering conditions and improve their performance as laser materials. Furthermore, we will investigate the polarization and thermo-optical properties of fluorapatite ceramics in order to evaluate their potential as high-power laser materials.

## Figures and Tables

**Table 1:** In-line transmittance at the wavelength of 1000 nm and average grain sizes of FAP ceramics prepared using different sintering temperatures.

Sintering Temperature (°C)	In-Line Transmittance at 1000 nm (%)	Average Grain Size (nm)
900	23.0	88
950	86.3	101
1000	86.4	134
1050	84.0	259
1100	86.1	528
1200	77.6	1644

**Table 2:** Physical properties of the FAP ceramics sintered at 1000 °C along with thermal shock parameter together with FAP and S-FAP single crystals.

Host Materials	*α* (×10^−6^ K^−1^)	*Κ* (W/mK)	*σ* (MPa)	*R_T_* (W/m)	Refs.
FAP ceramics	11.5	1.3	161	100	This work
Yb:S-FAP single crystal	8.4, 9.5	2.0	~102	125	[12]
FAP single crystal	10.0, 9.4	2.1, 1.9	—	—	[31,32]

## Data Availability

The data presented in this study are available upon request from the correspondence author.
